# Progression-free and overall survival in metastatic castration-resistant prostate cancer treated with abiraterone acetate can be predicted with serial C11-acetate PET/CT

**DOI:** 10.1097/MD.0000000000004308

**Published:** 2016-08-07

**Authors:** Jacob Farnebo, Agnes Wadelius, Per Sandström, Sten Nilsson, Hans Jacobsson, Lennart Blomqvist, Anders Ullén

**Affiliations:** a Department of Diagnostic Radiology and Nuclear Medicine, Karolinska University Hospital and Department of Molecular Medicine and Surgery; b Department of Oncology, Karolinska University Hospital and Department of Oncology and Pathology, Karolinska Institutet, Stockholm, Sweden.

**Keywords:** abiraterone acetate, C11-acetate PET/CT, metastatic castration-resistant prostate cancer

## Abstract

In this retrospective study, we evaluated the benefit of repeated carbon 11 (C11)-acetate positron emission tomography/computed tomography (PET/CT) to assess response in patients with metastatic castration-resistant prostate cancer (mCRPC) treated with abiraterone acetate (AA).

A total of 30 patients with mCRPC were monitored with C11-acetate PET/CT and PSA levels during their treatment with AA. Retrospective evaluation of their response was made after 102 days (median; range 70–155) of treatment. Statistical analyses were employed to detect predictors of progression-free survival (PFS) and overall survival (OS), and potential correlation between serum levels of PSA, standardized uptake values (SUVpeak), and bone lesion index measured from PET were investigated.

At follow-up 10 patients exhibited partial response (PR), 10 progressive disease (PD), and 10 stable disease (SD), as assessed by PET/CT. In survival analysis, both PR and PD were significantly associated with PFS and OS. CT response was also associated with OS, but only 19/30 patients demonstrated a lesion meeting target lesion criteria according to RECIST 1.1. No PET/CT baseline characteristic was significantly associated with PFS or OS. A PSA response (reduction in the level by >50%) could also predict PFS and OS. In the subgroup lacking a PSA response, those with PD had significantly shorter OS than those with PR or SD.

PFS and OS in patients with mCRPC treated with AA can be predicted from repeated C11-acetate PET/CT. This may be of particular clinical value in patients who do not exhibit a PSA response to treatment.

## 1 Introduction

The reported incidence of prostate cancer, one of the most common cancers in men worldwide, has increased during the last decade as a result of an aging population and more widespread testing of prostate-specific antigen (PSA).^[1]^ Although most of these patients with localized disease can be cured by surgery or radiotherapy, many relapse and eventually develop metastases. Since the discovery that androgen plays a key role in the growth of prostate cancer, androgen deprivation treatment (ADT) has been the primary therapy for metastatic prostate cancer.^[2]^ However, most patients receiving such treatment eventually progress to metastatic castration-resistant prostate cancer (mCRPC).

During the past decade several new treatments based on principally different underlying mechanisms, including cytotoxic compounds, radionuclides, vaccines- and hormonal approaches such as abiraterone acetate (AA), have been developed for patients with mCRPC.^[3]^ However, it is not yet known how to select the best treatment for a given individual or which sequence of treatments is optimal. In addition, evaluation of the efficacy of treatment remains a major clinical challenge. No reliable, validated biomarkers for personalized treatment are presently clinically available and the overall benefit is made on the basis of clinical benefits and radiological outcomes in combination with PSA and alkaline phosphatase (ALP) levels.

Administered orally, AA suppresses androgen synthesis by selectively inhibiting cytochrome P450-17-catalyzed α-hydroxysteroid dehydrogenase in testicular, adrenal, and prostatic tumor tissues. In the COU-AA-301 trial, AA together with prednisone prolonged overall survival (OS) by 4.6 months in comparison to placebo in patients with mCRPC previously treated with docetaxel.^[4]^ As a result AA is now one standard treatment option for this category of patients.

Metastases from CRPC are most commonly localized to the skeleton, with little or no involvement of soft tissue. According to the response evaluation criteria in solid tumors (RECIST 1.1),^[5]^ bone lesions without a soft tissue component are not considered to be measurable lesions. Accordingly, conventional cross-sectional radiological evaluation of the response of metastatic prostate cancer to treatment is of limited use. As reliable evaluation of the response to treatment is not always possible with computed tomography (CT), magnetic resonance imaging (MRI), or bone scintigraphy, the time required to reach an endpoint, rather than the endpoint itself, is often monitored during clinical trials on mCRPC.^[6]^ In this context, a combination of morphological and molecular imaging, such as positron emission tomography (PET) and CT (PET/CT), might be advantageous, as small molecular events often precede morphological alterations in the tumor. Unfortunately the tracer most commonly used by oncologists today, F18-fluoro-D-glucose, is not always optimally suited for imaging of prostate cancer, which grows slowly. Instead, more specific tracers reflecting elevated membrane synthesis or lipogenesis, such as radiolabeled choline or acetate, are more successful in this connection,^[7]^ especially for detecting early recurrence of prostate cancer following radiotherapy or surgery.^[8–10]^

Fatty acid synthase (FASN) plays a key role in lipogenesis, which is required in aggressive prostate cancers,^[11,12]^ where FASN expression of this enzyme is correlated to high Gleason scores.^[13]^ Visualization of lipid synthesis in cancer cells by C11-acetate PET/CT has been proposed as a surrogate biomarker for FASN activity,^[14]^ thus potentially providing a biomarker for evaluation of the response of patients with mCRPC to treatment, but to date only a few studies with PET/CT have addressed this possibility.^[15–19]^ Here, we hypothesized that the change in C11-acetate uptake as assessed by PET/CT imaging following treatment of mCRPC patients with AA is correlated to both the level of follow-up PSA and prognosis. Moreover, we examined whether this same approach can provide novel clinically relevant information concerning the prognosis for patients whose PSA levels were not lowered by AA treatment.

## 2 Patients and methods

### 2.1 Patients and treatment

During a 1-year period 35 consecutive patients at the Karolinska University hospital with mCRPC underwent C11-acetate PET/CT examinations before and during their treatment with AA. For this present retrospective evaluation of response 5 patients treated with AA for >14 days before PET/CT were excluded. Four patients included started their treatment in <1 week before the baseline PET/CT examination. Only a few days of treatment was considered to not have a significant impact in response assessment on the second PET/CT. Of the 26 remaining patients, baseline C11-acetate PET/CT examination was performed 4 days (median; range 0–63 days) before starting treatment. Thus, a total of 30 patients were included in the study. The baseline characteristics of our subjects are documented in Table 1.

**Table 1:**
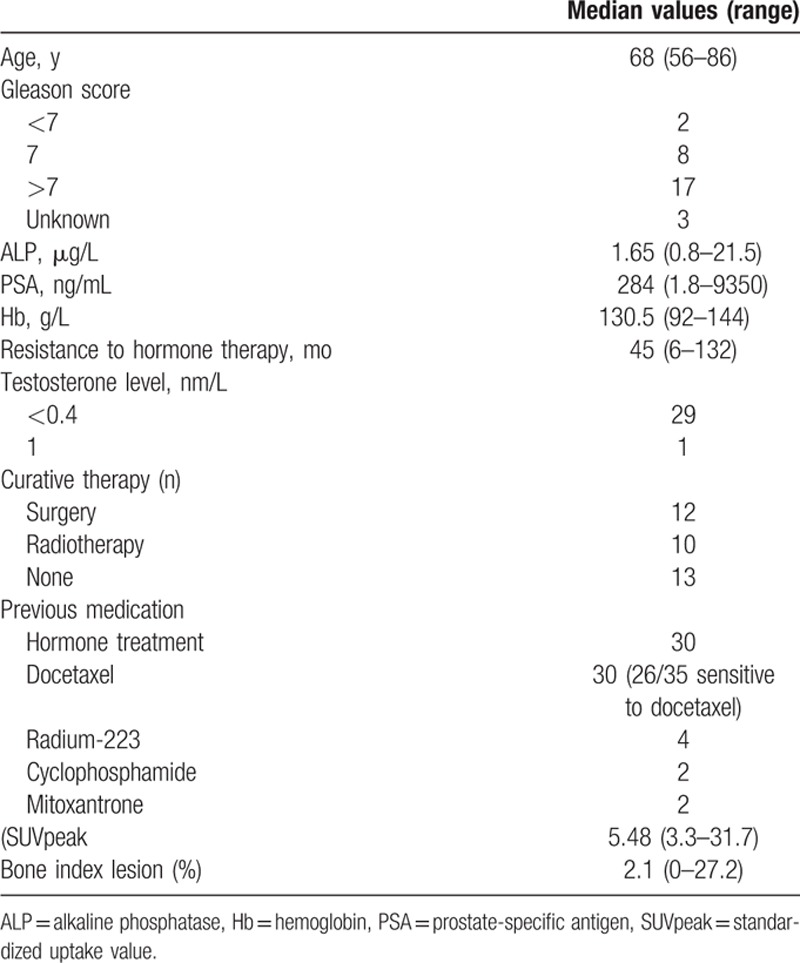
Baseline characteristics.

A second C11-acetate PET/CT examination was performed after 104 days of treatment (median; range 71–175) with 1000 mg AA (250 mg 4 times daily) and prednisolone 10 mg (5 mg 2 times daily). Approval to analyze the PET/CT examinations and available clinical information retrospectively was obtained from the regional ethical review board in Stockholm (Dnr 2015/1068-31).

### 2.2 Protocol for the C11-acetate PET/CT examination

All PET/CT examinations were performed using the same full-ring PET scanner (Biograph 64 TruePoint PET/CT scanner; Siemens Medical Solutions, Erlangen, Germany). PET-tracer uptake was initiated ∼23 minutes after injection of 600 to 700 MBq (16.2–18.9 mCi) of C11 acetate. The examination encompassed the inferior cervical neck to the proximal thighs. Acquisition time was 3 minutes per bed position during normal breathing. CT was performed as a full-tube current diagnostic examination without intravenous contrast enhancement using a continuous spiral 64-slice technique at 120 kV, pitch of 0.8, rotation speed of 0.5 seconds gantry rotation time, and a slice thickness of 1.2 mm. PET images were reconstructed with a CT-based attenuation correction algorithm provided by the manufacturer.

### 2.3 Assessment of response

An experienced radiologist (HJ) interpreted the C11-acetate uptake in all PET scans, as well as the morphological findings from the CT in a nonblinded fashion with access to clinical and previous information related to previous imaging. A clear elevation of C11-acetate uptake in metastatic lesions on follow-up examination compared to baseline was considered as progressive disease (PD), a lower as partial response (PR) and a similar level to be stable disease (SD). To account for variations in plasma clearance of C11 acetate and/or in the rate of uptake time by normal organs, C11-acetate uptake was adjusted to a tumor:liver ratio.

Another radiologist (JF) confirmed these interpretations and in case of any discrepancies a joint classification was made. In addition, at a later time point, the same radiologist also conducted evaluation of the CT part only (according to RECIST 1.1) and separate semiquantitative measurements of standardized uptake value (SUVpeak) according to Wahl et al^[20]^ on the metastatic lesion that exhibited most pronounced uptake. The retrospective evaluation of the PET/CT and the CT part of the PET/CT were carried out blinded to each other and to the prospective PET/CT interpretation. Metastatic lesions were selected for assessment only if the reviewer was certain of their malignancy, i.e., the lesion had a significantly high C11-acetate uptake or if the CT morphology of the lesion was obviously malignant.

**Figure 1:**
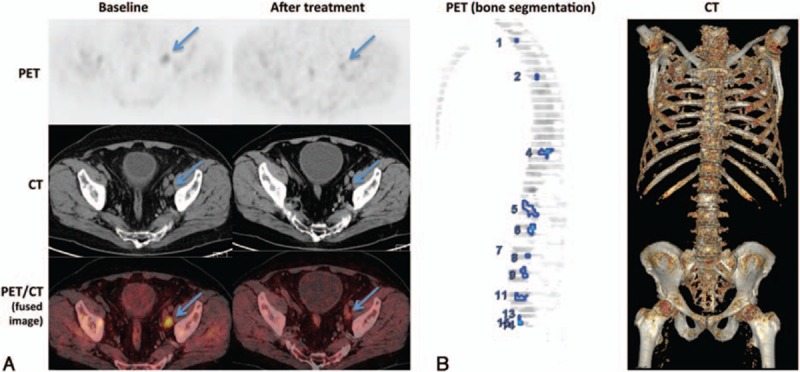
(A) PET/CT scans of a metastatic iliac lymph node before and after 3 months of treatment with abiraterone acetate. (B) Calculation of a bone lesion index by measuring the PET uptake volume with an automated segmentation algorithm and dividing this volume by the volume of the entire skeleton as determined by CT. PET/CT = positron emission tomography/computed tomography.

In a separate analysis of PET-positive metastatic bone lesions, an automated segmentation algorithm was applied to calculate an index representing the percentage of the skeletal mass occupied by metastases (Fig. 1). The combined volumes of all metastatic lesions with a SUV >3 and located in areas that attenuated >150 HU on the corresponding CT were divided by the volume of the entire skeleton. All semiquantitative analyses were performed with the PET VCAR software (GE Healthcare, Milwaukee, WI).

The PSA level in serum was measured at baseline and following 3 months of treatment. A PSA response was defined as a reduction >50%, PFS as the time to documentation of objective tumor progression/relapse, and OS as the number of days from the initiation of treatment until either death from any cause or the last date on which the patient was known to be alive (individuals censored in Kaplan–Meier curves).

### 2.4 Statistical analyses

To identify independent prognostic factors for outcome hazard ratios (HRs) and their 95% confidence intervals (CIs) were determined for each variable using the Cox univariate model of regression. The PFS and OS were also analyzed using the Kaplan–Meier method, and the log-rank test to assess any differences between outcome curves. The Spearman Rho test was used to look for potential correlations between continuous levels of PSA and the bone lesion index or SUVpeak. The Wilcoxon signed-rank test was utilized to compare the median differences between the PSA values at baseline and upon follow-up. Statistical analyses were conducted with the IBM SPSS statistics (version 22) software.

## 3 Results

The mCRPC patients in the present cohort were treated with AA for 266 days (median; range 89–876) and the length of this treatment was significantly correlated with OS (*P* = 0.001). Subsequently 19 patients received further treatment (13 cabazitaxel, 2 radium-223, 1 cabazitaxel and radium-223, 1 enzalutamide, 1 estramustine, and 1 with cyclophosphamide). Measured from baseline the PFS was 7 months (median; range 1–26) and OS 16.5 months (median; range 5–52).

### 3.1 Baseline PET/CT examination, biochemistry and associations with PFS and OS

According to the baseline PET/CT, 11/30 patients had bone metastases only, 2/30 had metastases only in lymph nodes, 13/30 had metastases in both bone and lymph nodes, and the remaining 4/30 patients had visceral metastases in addition to bone and/or lymph node metastases. In the evaluation of CT only from PET/CT, 19/30 patients demonstrated a measureable target lesion. The remaining 11 patients had no measureable target lesion but demonstrated sclerotic bone metastases.

The baseline PSA level was 284.5 ng/mL (median; range 1.8–9350 ng/mL) and demonstrated no association with PFS or OS (Table 2) upon univariate analyses. Indeed, none of the baseline characteristics examined was associated significantly with PFS or OS (Table 2). However, there was a significant correlation between the PSA level and bone lesion index (*P* = 0.002), but not between the same PSA level and SUVpeak of the lesion that showed most uptake (*P* = 0.067). The bone lesion index was not correlated to either PFS (*P* = 0.613) or OS (*P* = 0.518).

**Table 2:**
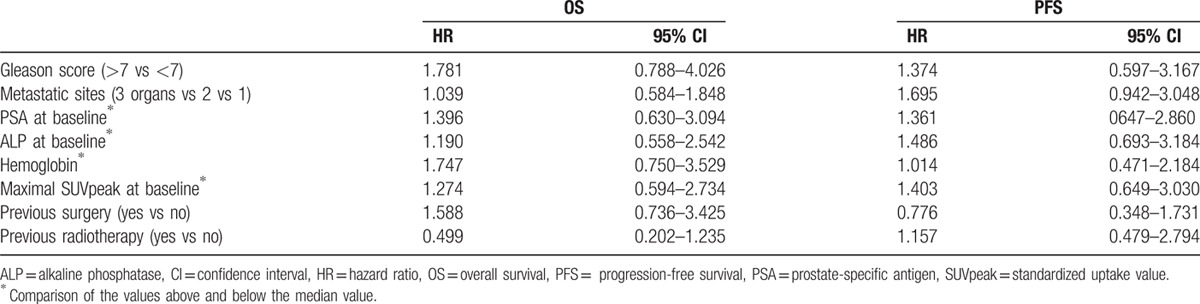
Univariate Cox regression analyses of potential associations between baseline characteristics and OS or PFS.

### 3.2 Follow-up PET/CT examination, biochemistry and associations with PFS and OS

**Figure 2:**
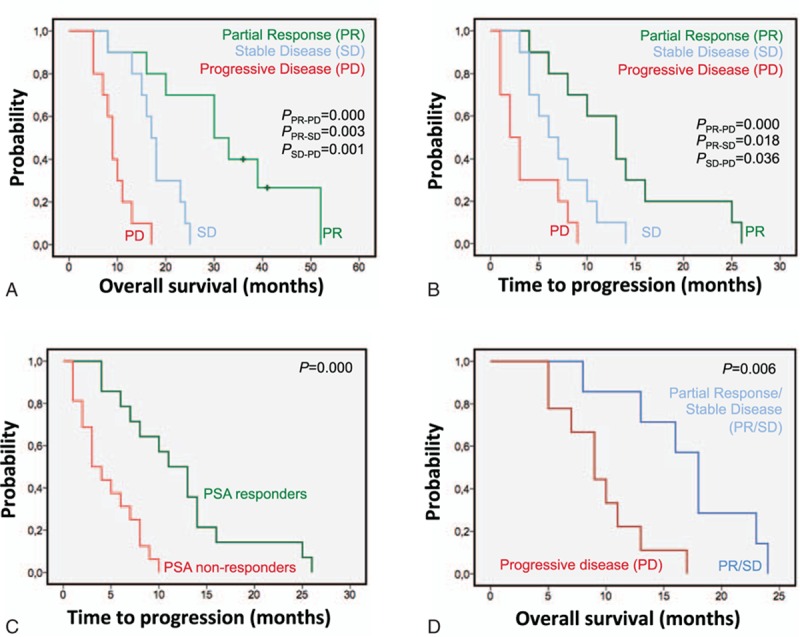
Kaplan–Meier survival curves (A and B) comparing patients demonstrating PR, SD, or PD as assessed by PET/CT, (C) comparing patients with PD and SD/PR as assessed by PET/CT in the subgroup of patients exhibiting no PSA response and (D) comparing patients demonstrating and lacking PSA response. PD = progressive disease, PET/CT =  positron emission tomography/computed tomography, PR = partial response, PSA = prostate-specific antigen, SD = stable disease.

According to the second PET/CT examination, 10 patients were classified as partial responders (PR), 10 as exhibiting SD, and 10 with PD. PET/CT response was associated with both PFS and OS as displayed in the Kaplan–Meier analysis (Fig. 2A and B). Semi-quantitative analysis of the lesion demonstrating most avid uptake upon follow-up revealed that bearing a lesion with higher SUVpeak is associated with shorter PFS and OS (Table 3). A reduction in ALP or elevation in the level of testosterone was not associated with OS or PFS (Table 3). The PSA level was significantly correlated to the bone lesion index (*P* = 0.004), but not to the SUVpeak of the most avid lesion.

**Table 3:**
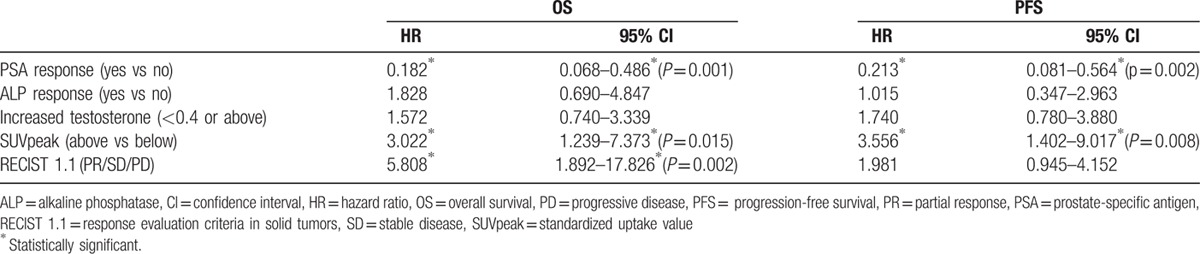
Univariate Cox regression analyses of potential associations between characteristics upon follow-up and OS or PFS.

At retrospective evaluation of CT only (according to RECIST 1.1) the corresponding distribution of response was 4/10/5 (PR/SD/PD) for the 19 patients demonstrating a target lesion at baseline. CT response was also significantly associated with PFS and OS (Table 3), but this result is questionable due to the large proportion patients with only nontarget lesions and small groups of PR and PD, which is reflected by a large confidence interval (Table 3). Evaluation of patients without target lesion at baseline was inconclusive and there was no documented progression of nontarget lesions.

The level of PSA at the time of follow-up was 158 (median; range 3–3190 ng/mL), which did not differ significantly from the baseline value (*P* = 0.088). Reductions by ≥50% were observed in 14 of the 30 patients (47%) and this PSA response was associated with PFS and OS (Table 3 and Fig. 2C). In addition, the PSA response was observed in all patients with radiological stable disease (PR or SD) except one. Moreover, in a subgroup analysis of patients lacking PSA response, there was a significant difference in OS between patients demonstrating a PD versus controlled disease (PR or SD) on PET/CT (Fig. 2D).

## 4 Discussion

In our cohort, a C11-acetate PET/CT examination followed by the same examination at a later time point was able to predict the clinical outcome in terms of PFS and OS, as was the level of PSA. Moreover, in our subgroup of clinically challenging patients demonstrating no PSA response, outcome was predicted by C11-acetate PET/CT indicating its complementary prognostic value in a clinical setting. At the same time, in connection with our baseline PET/CT examination, no variable could significantly predict outcome, indicating need for at least two examinations for prediction of outcome. The correlation between bone lesion index and PSA indicated that both reflected the total tumor burden in the skeleton, whereas neither turned out to be a good biomarker of outcome. Although computed tomography alone was associated with OS (but not PFS) it was not an optimal predictor of outcome in our cohort due to a large proportion (37%) of patients with only bone metastases (classified as nontarget lesions). Increased sclerosis on follow-up CT can be troublesome as interpretation can vary from response to progress depending on reviewer.

At present, the most applied biomarker of response in mCRPC is PSA, but in light of the expanding number of therapeutic options^[21]^ there is a growing interest in novel techniques for assessing response. Another advantage of PET/CT over PSA is that PET/CT provides anatomical information concerning the tumor burden that can be of value in explaining clinical symptoms or planning subsequent radiotherapy. Although a recent investigation showed that C11-acetate PET/CT and bone scintigraphy detect bone metastases equally well,^[22]^ most previous reports concerning C11-acetate PET/CT and prostate cancer have focused on early PSA relapse after prostatectomy or radiotherapy.^[8–10]^ To our knowledge, application of C11-acetate PET/CT for monitoring patients with mCRPC being treated with AA treatment has not been explored previously.

In 1998, Hara et al^[23]^ successfully showed that prostate cancer disease can be visualized with C11 choline and the first studies of C11 acetate on prostate cancer was published in 2002 by Oyama et al.^[24]^ In an intra-individual comparison between C11 choline and C11 acetate from 2003,^[25]^ the biodistribution between C11 choline and C11 acetate was shown to be similar. One obvious limitation with C11 is that it has half-life time of only 20.334 minutes, which requires radiosynthesis to be on-site and also to have an optimal PET imaging time in order to achieve adequate activity count rates for accurate diagnosis. For this reason F18-choline and Ga68/F18-prostate-specific membrane antigen (PSMA) with more desirable physical properties are emerging as feasible alternatives. Especially Ga68-PSMA has gained high attention with PSMA being significantly elevated on prostate cancer cells compared with in benign tissue.^[26]^ Studies have shown a higher diagnostic efficacy of Ga68-PSMA compared with F18 choline.^[27]^ However, prospective trials and clinical guidelines for Ga68-PSMA are still missing. Nonetheless, if uptake of C11 acetate reflects the levels of FASN in mCRPC, this might allow evaluation of the biochemical response of such tumors.^[14]^

The limitations to our study include the relatively small population of 30 patients and the fact that some of these received additional treatment before and/or after treatment with AA, which may have influenced the PFS and OS. We also observed that uptake of C11 acetate by liver tissue exhibited considerable case to case variation, which was adjusted for, although we do not know whether the pharmacokinetics of C11 acetate in the liver is similar to that in metastatic lesions. An additional limitation was the difference in time that elapsed between the baseline and follow-up PET/CT examinations. Four patients had already begun receiving AA when they underwent the baseline PET/CT. However, 2 of these were PR at follow-up (based on C11-acetate uptake) and the other 2 demonstrated a stable disease, indicating that the therapeutic effects before the baseline examination had been limited.

We conclude that repeated C11-acetate PET/CT examinations and PSA independent of each other can predict clinical outcome in patients with mCRPC treated with AA. For patients lacking a PSA response and thus with unclear benefit of the given treatment, C11-acetate PET/CT examinations add prognostic information that can be valuable in the subsequent treatment decision process. These results need to be validated in a larger study before being implemented in the clinical setting.
